# Draft Genome of the European Mouflon (*Ovis orientalis musimon*)

**DOI:** 10.3389/fgene.2020.533611

**Published:** 2020-11-19

**Authors:** Rui Su, Xian Qiao, Yun Gao, Xiaokai Li, Wei Jiang, Wei Chen, Yixing Fan, Bingwu Zheng, Yanjun Zhang, Zhihong Liu, Ruijun Wang, Zhiying Wang, Zhixin Wang, Wenting Wan, Yang Dong, Jinquan Li

**Affiliations:** ^1^College of Animal Science, Inner Mongolia Agricultural University, Hohhot, China; ^2^Key Laboratory of Animal Genetics, Breeding and Reproduction, Inner Mongolia Autonomous Region, Hohhot, China; ^3^Key Laboratory of Mutton Sheep Genetics and Breeding, Ministry of Agriculture, Hohhot, China; ^4^Engineering Research Center for Goat Genetics and Breeding, Inner Mongolia Autonomous Region, Hohhot, China; ^5^State Key Laboratory of Genetic Resources and Evolution, Kunming Institute of Zoology, Chinese Academy of Sciences, Kunming, China; ^6^NOWBIO Technology Co. Ltd, Kunming, China; ^7^College of Biological Big Data, Yunnan Agricultural University, Kunming, China; ^8^Yunnan Research Institute for Local Plateau Agriculture and Industry, Kunming, China; ^9^Daqingshan Wild Animal Park, Hohhot Gardens Management Bureau, Hohhot, China; ^10^Key Laboratory for Space Bioscience and Biotechnology, Center for Ecological and Environmental Sciences, Northwestern Polytechnical University, Xi’an, China

**Keywords:** mouflon, *Ovis orientalis musimon*, Illumina sequencing, *de novo* genome assembly, genus *Ovis*

## Abstract

Mouflon (*Ovis orientalis*) with its huge and beautiful horns is considered as one of the ancestors of domesticated sheep. The European mouflon (*Ovis orientalis musimon*) is in the Asiatic mouflon (*O. orientalis*) clade. In order to provide novel genome information for mouflon, moreover promote genetic analysis of genus *Ovis* both domestic and wild, we propose to sequence the mouflon genome. We assembled the highly heterozygous mouflon genome based on Illumina HiSeq platform using the next-generation sequencing technology. Finally, the draft genome we accessed approximately 2.69 Gb (42.15% GC), while N50 sizes of contig and scaffold are 110.1 kb and 10.4 Mb, respectively. The contiguity of this assembly is obviously better than earlier versions. Further analyses predicted 20,814 protein-coding genes in the mouflon genome and 12,390 shared gene families among bovine species. It is estimated that the divergence time between *O. orientalis musimon* and *Ovis aries* was 7.6 million years ago. The draft mouflon genome assembly will provide data support and theoretical basis for various investigations of the genus *Ovis* species in future.

## Introduction

Mouflon (*Ovis orientalis*) is a subspecies of the wild sheep with beautiful curved horns and red-brown coat color (Macdonald, 2001). It is considered to be one of the two ancestors for all modern domesticated sheep breeds (Hiendleder et al., 1998, 2002). This animal has been found in many different countries in Central Asia from Turkey in the west to Pakistan in the east (Hodges, 1997). Its usual habitat is in the mountainous areas up to 3,000 meters above sea level, but mouflon has also been introduced into European forested areas (IUCN Red List of Threatened Species, January, 2008, http://www.iucnredlist.org). Some previous studies considered the European mouflon (*Ovis orientalis musimon*) as a subspecies of Asiatic mouflon (*O. orientalis*; Corbet, 1986; Patterson et al., 1993; Rezaei et al., 2009). The survival of wild mouflon is threatened by habitat loss as a result of land reclamation, deforestation, mining activities, and expansion of human settlements (Ptak et al., 2002). It is also threatened by recreational hunters because of the highly prized horns (Garel et al., 2007). In addition, interbreeding with domesticated sheep resulted in the decreased number of genetically pure mouflon in the wild. Due to these factors, certain wild populations of the mouflon are listed as vulnerable by IUCN. This study reports a draft genome assembly of mouflon, and it will lay the ground work for further gene function annotating of the mouflon genome and revealing its evolutionary status, and comparative genomics, as well as promote the conservation of the mouflon and the breeding of genus *Ovis* in the future.

## Results

### Whole-Genome Sequencing of the Mouflon Genome on an Illumina Platform

A total of 780.6 Gb raw data were generated (Supplementary Table 1) from Illumina HiSeq 4000 platform (Illumina, CA, United States). The average GC content is 45.35%. In the quality control step, we filtered out low quality and repetitive data, and finally got 660.7 Gb of clean data for subsequent analysis (Supplementary Tables 1, 2).

### Estimation of the Mouflon Genome Size

The analysis result of all 21-mer sequences from quality-filtered reads was calculated and explained as a frequency graph (Supplementary Figure 1). Two distinct peaks were observed at the depth of 49× and 97×, respectively. The first peak indicated that the mouflon genome may have high heterozygosity. The heterozygosity ratio is 0.42%, belonging to the high heterozygosity ratio genome calculated by GCE (Liu et al., 2013). The repeat ratio is 41.66%. Estimation result of mouflon genome size was 2.87 Gb (Supplementary Table 3). The sequencing depth of clean data is about 230× genome coverage. Based on this result, we used Platanus to assemble this genome in the next step.

### Hybrid *de novo* Genome Assembly and Evaluation of Mouflon

Platanus (v. 1.2.1; Kajitani et al., 2014) were used to assemble HiSeq reads. Both paired-end (short-insert 244 bp∼700 bp) and mate pair (long-insert 1 kb∼15 kb) libraries were constructed according to the standard manufacturer’s instructions from Illumina (San Diego, CA, United States). Final draft mouflon genome of 2.69 Gb was assembled (42.15% GC), with contig and scaffold N50 size of 110,147 bp and 10,434,335 bp, respectively, (Table 1). BUSCO analysis identified 95.8% complete and 1.9% fragmented of 2,586 expected genes, respectively, (Supplementary Table 4). To assess base-level accuracy, integrity, and continuity of the genome assembly, Illumina reads from high-quality short-insert libraries were mapped to the assembled mouflon genome (Supplementary Table 5). The result showed that the total mapping ratio of all the 13 short-insert size libraries are up to 99.30%, indicating that our assembly results are of high quality.

**TABLE 1:** Statistics summary of the Platanus assemble results.

	**Contig**		**Scaffold**	
	**Size (bp)**	**Number**	**Size (bp)**	**Number**
N90	21,333	27,123	2,957,008	253
N80	39,976	18,219	5,114,525	185
N70	60,012	12,826	6,531,856	140
N60	83,112	9,071	8,527,209	102
N50	110,147	6,293	10,434,335	74
Longest	2,090,923		45,780,817	
average size	22,959		40,271	
Total number		66,243		18,131
(≥1000 bp)				
Total		115,470		66,744
Total size	2,651,188,186		2,687,907,747	
GC content (%)			42.15	

### Repeat Annotation of the Mouflon Genome Assembly

Tandem Repeats Finder, *de novo* prediction approaches and homology to Repbase sequences were combined to annotate repetitive sequences and TEs of mouflon (Supplementary Table 6 and Supplementary Figures 2, 3). Comprehensive analysis results showed that repeated sequences accounted for 45.90% of the mouflon genome assembly and the total length was about 1245.6 Mb (Supplementary Table 7).

### Gene Prediction and Non-Coding RNA Annotation

Multiple gene prediction methods were used to annotate protein-coding genes in the mouflon genome, including homology-based and *de novo* predictions. The analyses showed that 20,814 protein-coding genes were annotated in the mouflon genome with an average coding DNA sequence length of 1642 bp (Table 2 and Supplementary Figure 4). The final non-coding RNA annotation results include 482 miRNAs, 535 tRNAs, 114 rRNAs, and 1,428 snRNAs (Supplementary Table 8).

**TABLE 2:** General statistics of predicted protein-coding genes.

**Gene set**		**Number**	**Average gene length**	**Average CDS length**	**Average exon number**	**Average exon length**
Homolog	*Capra hircus*	24,058	25,496.561	1,415.625	8.058	175.690
	*Homo sapiens*	25,924	22,837.589	1,283.671	7.310	175.601
	*Mus musculus*	24,598	23,055.830	1,315.210	7.446	176.643
	*Ovis aries*	25,151	22,871.331	1,325.002	7.722	171.597
*Denovo*	AUGUSTUS	21,653	45,863.010	1,463.875	8.665	168.933
	Genscan	43,572	40,224.966	1,277.758	7.697	166.014
	GlimmerHMM	120,362	9,294.303	628.566	2.866	219.354
EVM		20,814	46,648.408	1,641.787	9.941	165.145

### Gene Family Clustering Analysis

In order to discern and predict the amount of potential orthologous gene families, gene sequences of *O. orientalis musimon*, *Bison* (GCA_000754665.1 Bison_UMD1.0), *Bos mutus* (GCA_000298355.1 BosGru_v2.0), *Bubalus* (GCA_000471725.1 UMD_CASPUR_WB_2.0), *Camelus ferus* (GCA_000311805.1 CB1), *Capra hircus* (GCA_001704415.1 ARS1), *Odocoileus virginianus* (GCA_002102435.1 Ovir.te_1.0), *Ovis aries* (GCA_000298735.2 Oar_v4.0), *Pantholops hodgsonii* (GCA_000400835.1 PHO1.0) were downloaded from National Center for Biotechnology Information (NCBI)^1^ and *Homo sapiens* (GCA_000001405.22 GRCh38.p7) were downloaded from Ensembl Genome Browser^2^. Among the total of 13,987 mouflon gene families, 140 (1%) appear to be lineage specific. There are 12,390 (88.6%) gene families shared among *O. orientalis musimon*, *Bison*, *Bubalus*, *O. aries*, and *C. hircus*. The total of 13,400 gene families were shared between *O. orientalis musimon* and *O. aries* as well as 350 gene families were specific to both (Supplementary Table 9 and Supplementary Figures 5, 6). Synteny analysis results show that the relationship of variations between mouflon and sheep genome (Supplementary Figure 7). The left part from outer circle to the inner circle, represent the mouflon scaffold, gene density and repeat sequence density at the corresponding position, respectively, while the right part represents chromosome of sheep, as well as the line between them represents the synteny relationship.

### Phylogenetic Tree Construction and Divergence Time Estimation

The result of phylogenetic tree showed that *O. orientalis musimon* was more closely related to *C. hircus* than *P. hodgsonii* in the Caprinae family (Supplementary Figure 8a). It is estimated that the divergence time between *O. orientalis musimon* and *O. aries* was 7.6 million years ago (Supplementary Figure 8b).

### Analyses of Gene Family Expansion and Contraction

We identified 177 expanded gene families and 2047 contracted gene families in the mouflon genome, which is special among those species in Caprinae (Supplementary Figure 9). The expanded gene families were enriched in 35 GO terms including binding, Cellular Component, etc. (Supplementary Figure 10). We further used the free ratio model to calculate the average Ka/Ks values and the branch-site likelihood ratio test to identify positively selected genes (PSGs). A total of 165 PSGs were identified in the mouflon genome (Supplementary Table 10). The result of KEGG pathway analysis showed that 78 of the PSGs were enriched in 164 pathways, and 7 of these pathways were significantly enriched (*P* < 0.05; Supplementary Table 11).

### PSMC Analysis of Effective Population Sizes

According to the results of Pairwise Sequentially Markovian Coalescent (PSMC) analysis, the effective population size (Ne) of the mouflon shows two peak at ∼1 millions of years ago (Mya) and ∼120 thousand years ago (Kya), followed by 3 distinct declines, and 2 distinct increases.

## Discussion

According to the result of KEGG pathway analysis, the PSGs can be divided into 2 groups. The first group is about nutrition and metabolism (Protein digestion and absorption, AGE-RAGE signaling pathway in diabetic complications and Caffeine metabolism). It’s very interesting that we found xanthine dehydrogenase (*XDH*) enriched in the Caffeine metabolism pathway is related to the milk production of sheep and goat, especially affecting the formation of Lipid droplets (Suárez-Vega et al., 2016; Toral et al., 2016). The second group is about signal transduction (cGMP – PKG signaling pathway, Oxytocin signaling pathway, ECM-receptor interaction and Renin secretion). It is worthwhile to note that some important genes are involved in different pathways at the same time, which need more attention in future studies. For example, nuclear factor of activated T-cells 5 (*NFAT5*) is important in both immune response and high osmotic stress pathways (Bounedjah et al., 2012). Calcium voltage-gated channel subunit alpha1 F (*CACNA1F*) and calmodulin 2 (*CALM2*) were involved in three signal transduction pathways at the same time. *CACNA1F* encodes a multipass transmembrane protein which acts as an alpha-1 subunit of the voltage-dependent calcium channel, mediating the influx of calcium ions into the cell (Fisher et al., 1997; Strom et al., 1998). Mutations in *CACNA1F* are reported to be associated with type 2 congenital stationary night blindness (CSNB2; Men et al., 2017), cone-rod dystrophy 3 (CORDX3; Hauke et al., 2013), Aland island eye disease (AIED; Weleber et al., 1989), and X-linked retinitis pigmentosa (XLRP; Zhou et al., 2014). *CALM2* is a member of the calmodulin gene family; increased *CALM2* mRNA levels may reflect an important role for calmodulin in expansion-induced fetal lung growth of sheep (Gillett et al., 2002). We also found some PSGs related to Lipid metabolism (*APOL3, ADARB2*, and *AP2B1*). Apolipoprotein L3 (*APOL3*) belongs to the apolipoprotein L gene family and has the function of biochemical metabolism by carrying lipids (Duchateau et al., 2001). Adenosine deaminase, RNA specific B2 (*ADARB2*) related to longevity is associated with metabolic disorders like the abdominal circumference, body mass index, serum triglyceride level, and serum adiponectin level (Oguro et al., 2012). Adaptor related protein complex 2 subunit beta 1 (*AP2B1*) was reported to be associated with milk yield and fat yield traits (Kolbehdari et al., 2009; Chen et al., 2018), as well as meat quality traits (Piórkowska et al., 2018). To a certain extent, these PSGs reflect the adaptation process of wild sheep domestication to environmental changes.

Population contraction started from 1 Mya, and reached the bottleneck at ∼400 Kya. After 1 Mya, the longer glacial cycle and the cold climate might affect the reduction of the populations (Muller and Macdonald, 1997). This bottleneck of the population may be related to the Penultimate Glaciation (Zheng et al., 2002). As the glaciers receded, the temperature gradually increased and the population began to expand and reached the second flourishing period in 120 Kya which may coincide with warm Eemian interglacial period that genetic diversity of species is enriched (Pilot et al., 2006; Hailer et al., 2012). From ∼120 Kya to the present the Ne has continued to decline, with a short plateau period between ∼400 Kya and ∼900 Kya. The most recent decline involved a decrease of Ne at least a 9-fold, and it happened about ∼40 Kya (Supplementary Figure 11). This process corresponded to the abrupt change of climate after the last glaciations (Cooper et al., 2015).

It is well known that N50 size of contig and scaffold are important indicators of assembly integrity and quality. Compared with the earlier version of mouflon genome assembly Oori1 with contig and scaffold N50 size of 39,721 bp and 2,217,029 bp, respectively^3^, our result shows better contiguity and quality. Although this assembly vision is only based on scaffold level, the N50 size is also larger than the newly published Marco Polo Sheep (*Ovis ammon polii*) genome assembly with contig and scaffold N50 size of 30,772 bp and 5,492,388 bp, respectively, (Yang et al., 2017; Supplementary Table 12). The significant improvement of contig has some effects on better gene annotation and the improvement of scaffold is very helpful to the improvement of genome integrity, which revealed that the new version of mouflon genome we assembled was with high quality. In the future we still need to update our sequence technology to upgrade mouflon genome assembly to chromosome level.

## Conclusion

In conclusion, the mouflon genome sequencing, assembly, annotation, and evolutionary analysis were reported in this study. This genome assembly will provide valuable resources for the further genetic research of mouflon, and might establish a theoretical basis for the conservation of genetic resources and sheep evolution in the future.

## Materials and Methods

### Sample Processing and Whole-Genome Sequencing

Genomic DNA was extracted from the venous blood of a single (sex unknown) European mouflon (Daqingshan Wild Animal Park, Hohhot, Inner Mongolia, China) with the Tiangen Blood Genomic DNA Extraction Kit (Tiangenbiotech, Beijing). Paired-end libraries with short-insert sizes from 244 bp to 700 bp were constructed with NEBNext Ultra II DNA Library Prep Kit for Illumina (NEB, United States), while mate pair libraries with long-insert sizes from 1 kb to 15 kb were constructed with Illumina Nextera Mate Pair Library Preparation Kit (Illumina, United States; Supplementary Table 5). Sample processing was according to the manufacturer’s instructions. Illumina HiSeq 4000 platform (Illumina, CA, United States) were used for sequencing with the PE-150 module (Quail et al., 2012). Then the correction steps were performed with SOAPec_v2.01 (Luo et al., 2012) using the parameters: −k 21, −i 450,000,000.

### Estimation of the Mouflon Genome Size

SOAPec_v2.01 was used for 21-mer frequency distribution analysis with quality-filtered reads from the Illumina platform. All 21-mer sequences were extracted from paired-end reads from short-insert size libraries (<1 kb) that passed quality control, and the frequency of each 21-mer was calculated and plotted. The heterozygosity ratio of genome was calculated by GCE (Liu et al., 2013). The genome size was estimated by the formula Genome_Size = #_Total_Kmer/Expected_Kmer_Depth.

### Hybrid *de novo* Genome Assembly and Evaluation of Mouflon Genome

Both short-insert and long-insert libraries sequenced on Illumina HiSeq 4000 platform were used for hybrid *de novo* genome assembly. HiSeq reads were assembled using Platanus (v. 1.2.1; Kajitani et al., 2014) with parameters “−k 47 −s 10 −d 0.3 −u 0.1,” for contigs and “−s 47 −v 34 −l 2,” for scaffolds. The completeness of the final assembly were evaluated using Benchmarking Universal Single-Copy Orthologs (BUSCO; v.2.0; Simão et al., 2015) with the mammal gene set. To assess the assembly quality, BWA and SAMtools (Li et al., 2009) were used to map Illumina reads for short-insert size libraries with high quality onto the mouflon genome assembly.

### Repeat Annotation of the Mouflon Genome Assembly

Tandem Repeat Finder (v. 4.07b) was used to search the mouflon genome for tandem repeats (Benson, 1999) with default parameters. Transposable elements (TEs) were firstly predicted in the genomes by homology searches to known RepBase TE libraries (Jurka et al., 2005) using RepeatMasker (v. 3.3.0) and RepeatProteinMask (Chen, 2004) with default parameters. A *de novo* repeat library was constructed using RepeatScout. We obtained consensus sequences and classification information from each repeat family. RepeatScout consensus sequences were used as an input library in order to search for repetitive elements in the assembled genome using RepeatMasker with default parameters.

### Gene Prediction

Protein-coding genes in the mouflon genome were annotate by multiple methods like homology-based predictions and *de novo* predictions. In homology-based prediction, proteins from *C. hircus*, *H. sapiens*, *Mus musculus*, *O. aries* were obtained from the NCBI, and mapped to the mouflon genomes using TBLASTN analysis with a cutoff *E*-value of 1e^–5^. Then, homologous regions defined by TBLASTN were fed into GeneWise (v. 2.2.0; Birney and Durbin, 2000) to obtain gene models for predicting the gene structure contained in each protein region. BLAST hits corresponding to reference proteins were concatenated by Solar (v. 0.9.6; Beijing Genomics Institute; Li et al., 2016). After removing low-quality records, the genomic sequences of each reference protein were extended upstream and downstream by 2000 bp aimed to represent the protein-coding region. For *de novo* prediction, AUGUSTUS (v. 2.5.5; Stanke et al., 2006), GENSCAN (v. 1.0; Cai et al., 2014), and glimmerHMM (v. 3.0.2; Majoros et al., 2004) were employed to predict coding genes. Evidence Modeler (released 25 June 2012) was used to combine the homology and *de novo* predicted gene sets and creat a comprehensive and non-redundant reference gene set.

### Non-Coding RNA Annotation

For tRNA annotation step, tRNAscan-SE (v.1.3.1; Lowe and Eddy, 1997) was used with default parameters for eukaryotes. Homology-based rRNA was annotated by BLASTN which mapping rRNAs to the mouflon genome, with parameters of “*E*-value = 1e^–5^.” INFERNAL (v.1.1; Nawrocki et al., 2009) was used to predict miRNA and snRNA genes the with Rfam database (release 11.0; Gardner et al., 2011).

### Gene Family Clustering Analysis

We use standard settings (BLASTP *E*-value < 1e^–5^) to apply the OrthoMCL (v.2.0.9) pipeline (Li et al., 2003) for the calculation of all-against-all similarities among *O. orientalis musimon*, *Bison*, *B. mutus*, *Bubalus*, *C. ferus*, *C. hircus*, *O. virginianus*, *O. aries*, *P. hodgsonii*, and *H. sapiens*, in order to discern and estimate the amount of potential orthologous gene families. Genes were then clustered into gene families using Hcluster_sg with consideration of proteins of out-group species (*Homo sapiens*). Gene sequences were all downloaded from NCBI (see text footnote 1, respectively). Synteny analysis were carried out to identified the variations between mouflon and sheep genome using the program LAST (LAST,RRID:SCR_006119; Kiełbasa et al., 2011).

### Phylogenetic Tree Construction and Divergence Time Estimation

Treefam was used in the gene family clustering analysis to identify all 1006 single-copy orthologous genes from the *O. orientalis musimon*, *Bison*, *B. mutus*, *Bubalus*, *C. ferus*, *C. hircus*, *O. virginianus*, *O. aries*, *P. hodgsonii*, and *H. sapiens*. Mrbayes was used to construct a phylogenetic tree. The species divergence time was estimated based on four-fold degenerate sites via Bayesian relaxed molecular clock (BRMC) approach using the program MULTIDIVTIME as implemented in the Thornian Time Traveler (T3) package^4^. The divergence time of each node was estimated by the PAML (PAML, RRID:SCR 014932) MCMCtree program v4.5 and calibrated against the timing of the divergence refer to http://www.timetree.org/.

### Analyses of Gene Family Expansion and Contraction

According to the calculated phylogeny and the divergence time, we used CAFE (v. 2.1; De et al., 2006) to search gene families that under expansion and/or contraction in *O. orientalis musimon*, *Bison*, *B. mutus*, *Bubalus*, *C. ferus*, *C. hircus*, *O. virginianus*, *O. aries*, and *P. hodgsonii*. The parameters were “*P* = 0.05, number of threads = 4, number of random = 10000, and search for lambda (λ).” CAFE can estimate the global birth and death rate of gene families, infer the most likely gene family size at all internal nodes, identify gene families that have accelerated rates of gain and loss (quantified by a *p*-value) and identify which branches cause the *p*-value to be small for significant families. This method estimated the family sizes in the common ancestor, and then defined expansion and contraction by comparing the family size between the current species and the ancestor. We calculated the ratio of non-synonymous substitution rate over synonymous substitution rate (Ka/Ks), which can be used as an indicator of selective pressure acting between all the protein-coding genes. Comparisons of homologous genes with a high Ka/Ks ratio are usually said to be evolving under positive selection, so that we call these genes are PSGs. Ka/Ks were estimated using the software KaKs_Calculator, with the method of model averaging (GMYN). We choose the result with Ka/Ks more than 1(Fisher’ test, *P* < 0.05), and blast identity more than 80%.

### PSMC Analysis of Effective Population Sizes

In the final analysis, the demographic history of the mouflon were inferred with the PSMC model (Li and Durbin, 2011). PSMC modeling was done by bootstrapping approach, and the variance of the simulated results were estimated with sampling performed 100 times. We used SAMtools v0.1.19 to obtain Consensus sequences (Li et al., 2009) and the data was divided into non-overlapping 100-bp bins. The analysis parameters were −N25 −t15 −r5 −p “4 + 25 × 2 + 4 + 6.” We removed sites at which the root-mean-square mapping quality of reads covering the site was below 25, the inferred consensus quality was below 20, and read depth was either more than twice or less than one-third of the average read depth across the genome assembly. After filtering, we obtained a diploid consensus genome sequence for the PSMC analysis.

## Data Availability Statement

The sequencing reads of each sequencing library have been deposited at NCBI with the Project ID SRP106760. The SRA accession number for the sequenced individual (Sample ID: SAMN06909321) is SRS2175019.

## Ethics Statement

All animal procedures in this paper were approved by the Inner Mongolia Agriculture University Animal Care and Use Committee in accordance with the National Animal Care Standard (GB 14925-2010). All experiments were performed in accordance with relevant guidelines and regulations. All efforts were made to minimize animal suffering.

## Author Contributions

RS, YD, and JL designed the study. YG assembled the genome and analyzed the data. XQ, RS, YG, and WC participated in manuscript revision. WJ did samples collection. BZ provided the samples. XQ, XL, YF, and WJ extracted DNA. WW constructed libraries. YZ, ZL, RW, ZYW, and ZXW conceived the overall study and revised the manuscript. All authors read and approved the final manuscript.

## Conflict of Interest

YG was employed by the company NOWBIO Technology Co. Ltd., Kunming, Yunnan, China. The remaining authors declare that the research was conducted in the absence of any commercial or financial relationships that could be construed as a potential conflict of interest.

## Footnotes

1 ftp://ftp.ncbi.nih.gov/genomes

2 http://oct2016.archive.ensembl.org

3 https://www.ncbi.nlm.nih.gov/assembly/GCF_000765115.1

4 ftp://abacus.gene.ucl.ac.uk/pub/T3/

## Abbreviations

IUCN: International Union for Conservation of Nature and Natural Resources
BUSCO: Benchmarking Universal Single-Copy Orthologs
TEs: Transposable elements.
