# Supplementary material for: Draft Genome of the European Mouflon (Ovis orientalis musimon)
Source: Front Genet. 2020 Nov 19;11:533611. doi: 10.3389/fgene.2020.533611 (PMC7710762; doi:10.3389/fgene.2020.533611)
Supplement: Supplementary file 1 [file Data_Sheet_1.zip › Supplementary Figures & Tables.DOCX]

**Supplementary Files**


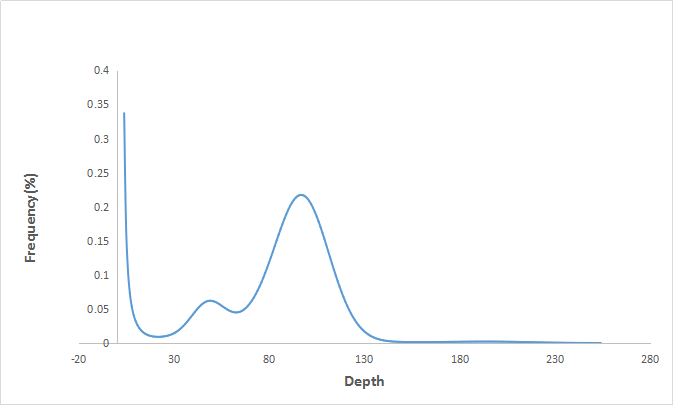


**Supplementary Figure 1. 21-mer frequency distribution of sequencing reads.**


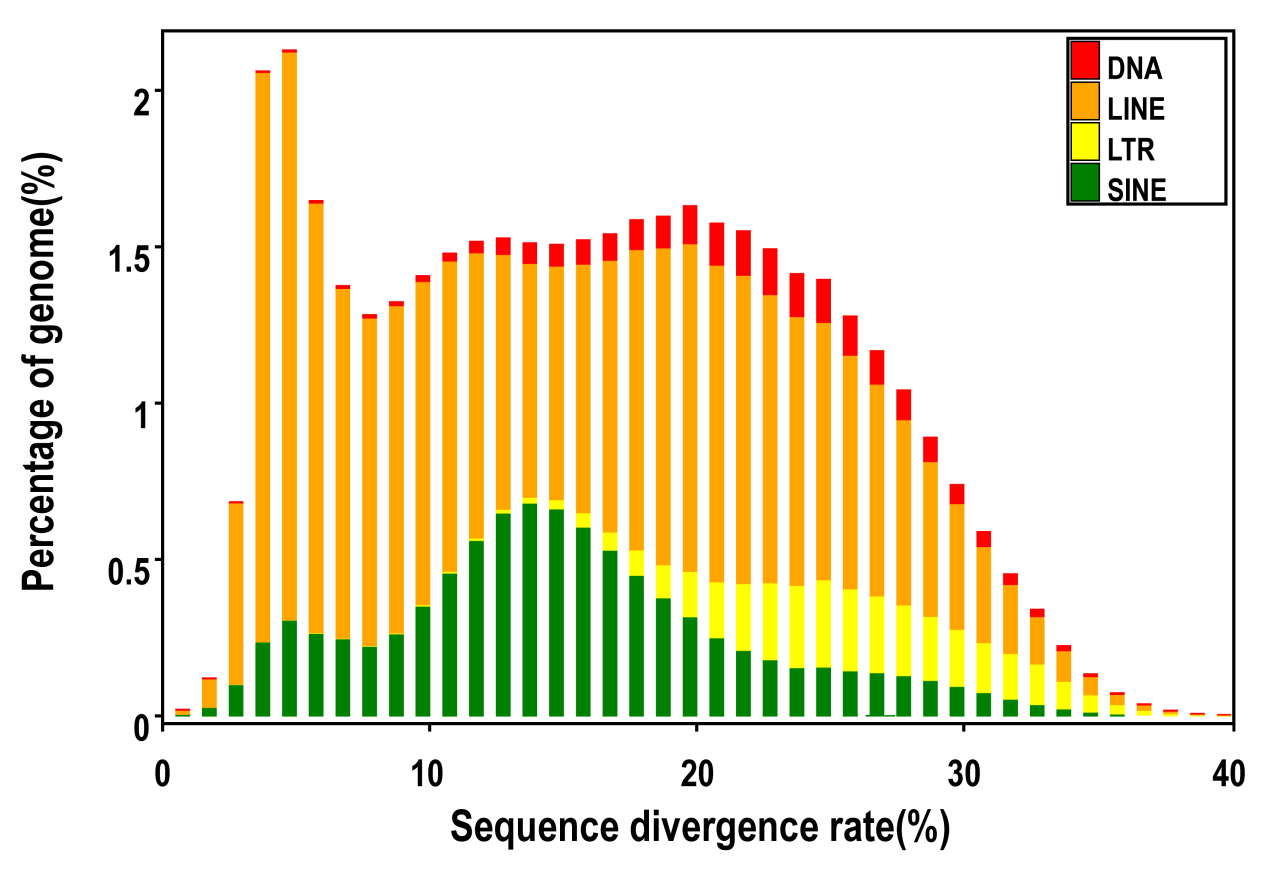


**Supplementary Figure 2. Distribution of Divergence Rate of each Type of mouflon’s TE.** The divergence rate was calculated between the identified TE elements in the genome by homology-based method and the consensus sequence in the Repbase.


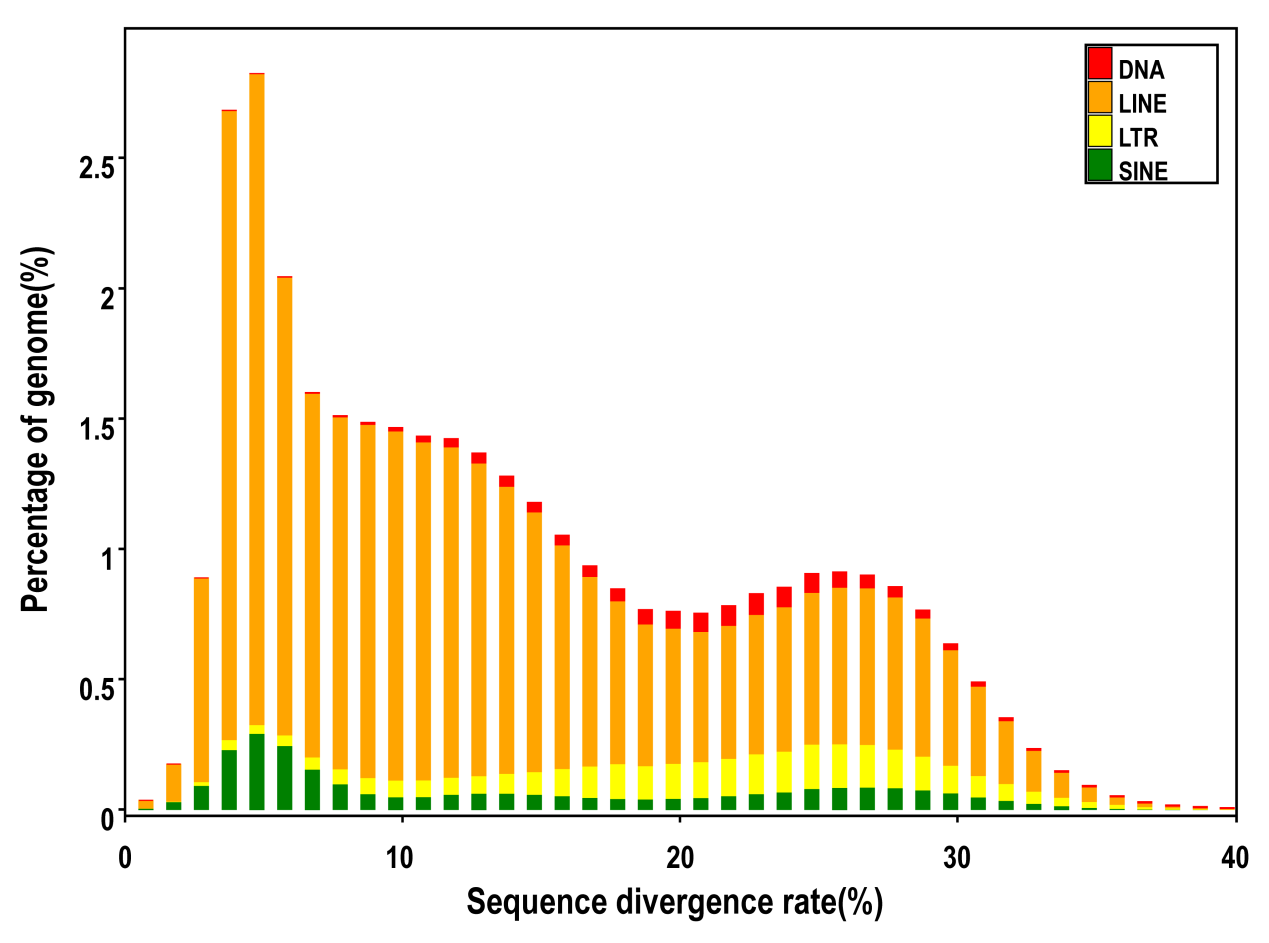


**Supplementary Figure 3. Distribution of Divergence Rate of each Type of mouflon’s TE.** The divergence rate was calculated between the identified TE elements in the genome by de novo method and the consensus sequence in the predicted TE library.


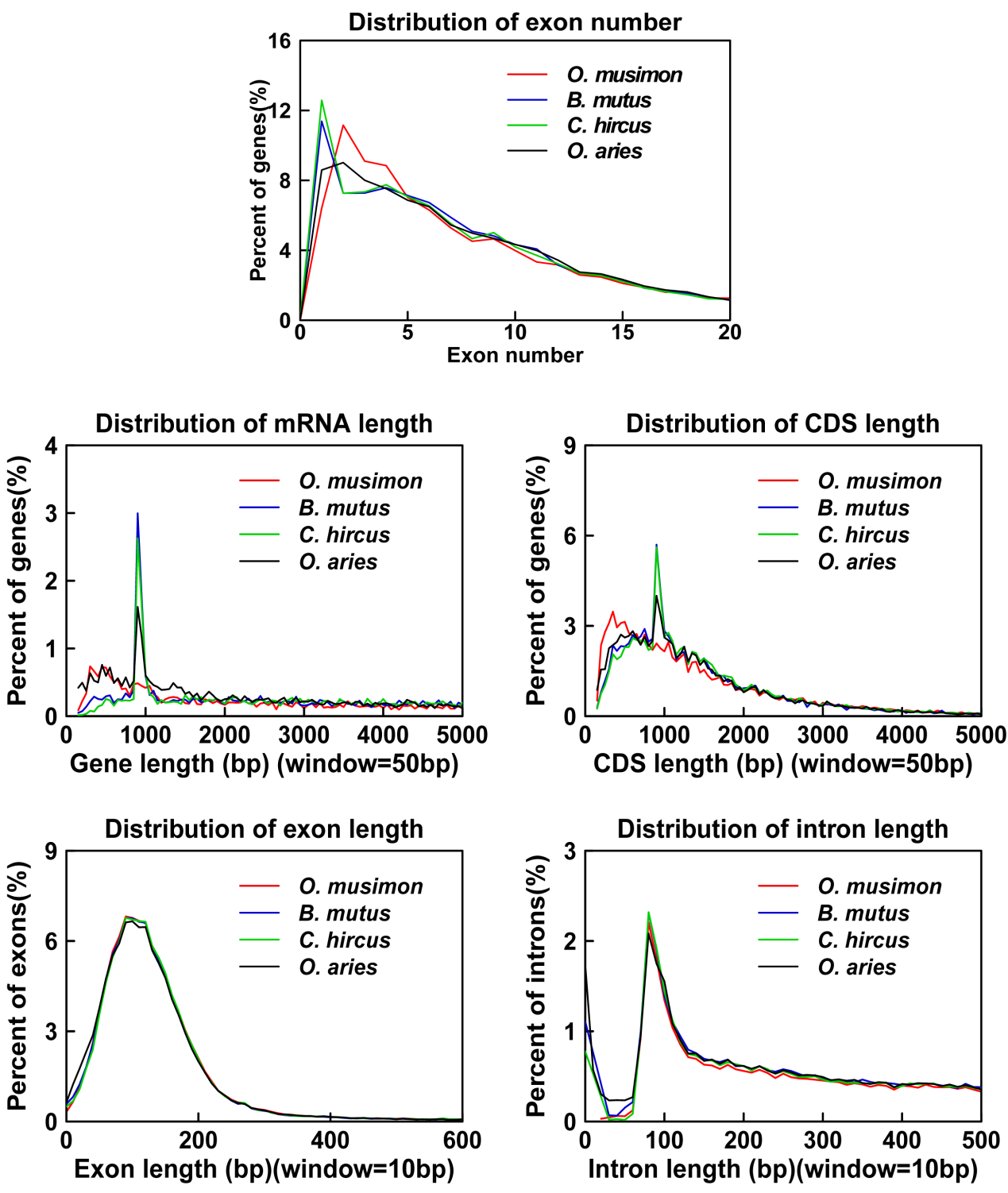


**Supplementary Figure 4. Comparisons of gene parameters among** ***Ovis orientalis musimon (O. musimon), Bos mutus (B. mutus), Capra hircus (C. hircus), Ovis aries (O. aries) genomes*.**


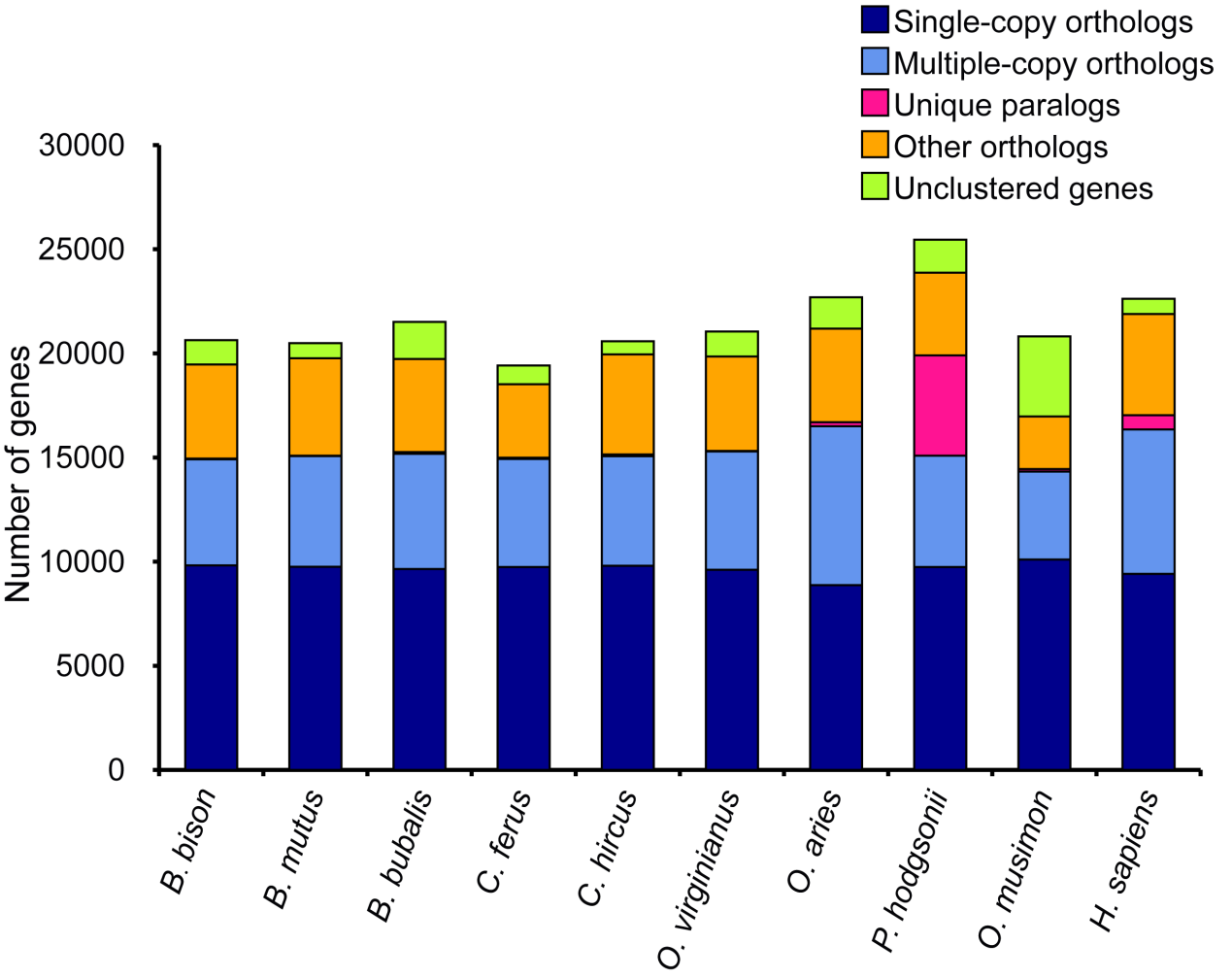


**Supplementary Figure 5. Statistics of orthologous families for *Ovis orientalis musimon (O. musimon),*** ***Bison (B. bison),*** ***Bos mutus (B. mutus),*** ***Bubalus (B. bubalis),*** ***Camelus ferus (C. ferus),*** ***Capra hircus (C. hircus),*** ***Odocoileus virginianus (O. virginianus),*** ***Ovis aries (O. aries),*** ***Pantholops hodgsonii (P. hodgsonii),* and** ***Homo sapiens (H. sapiens )*.**


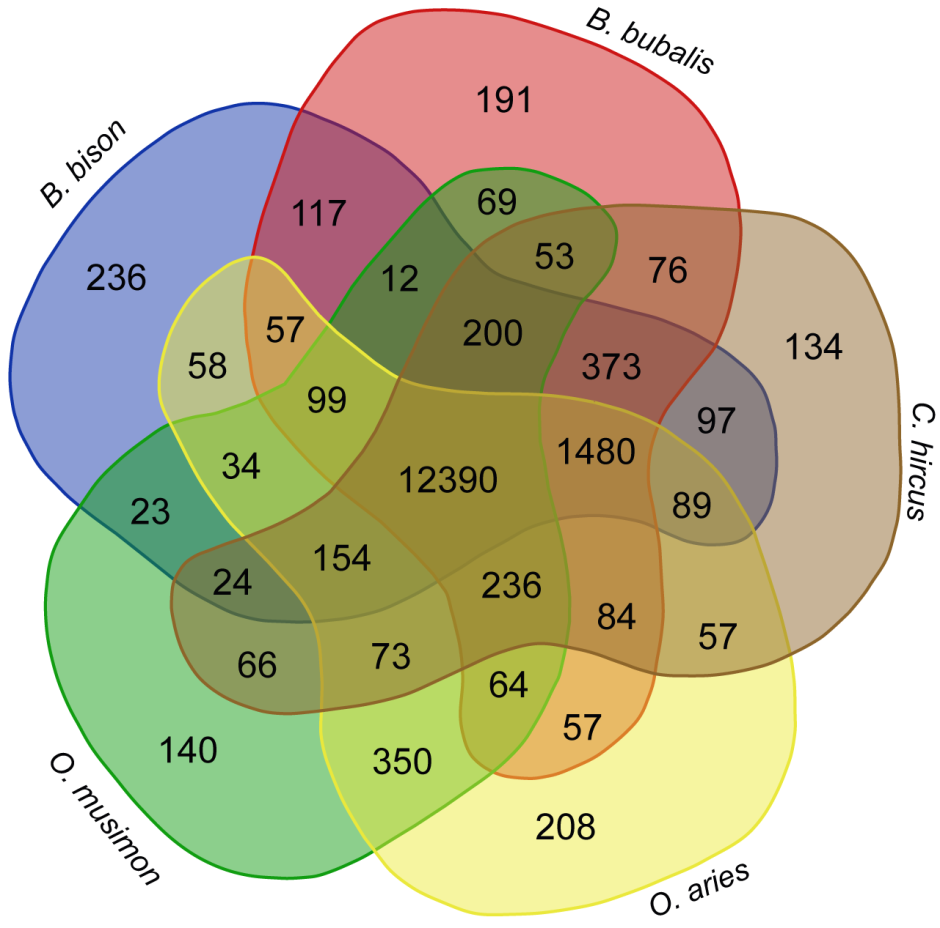


**Supplementary Figure 6.** **Venn diagram showing unique and shared gene families among five sequenced species.**

**
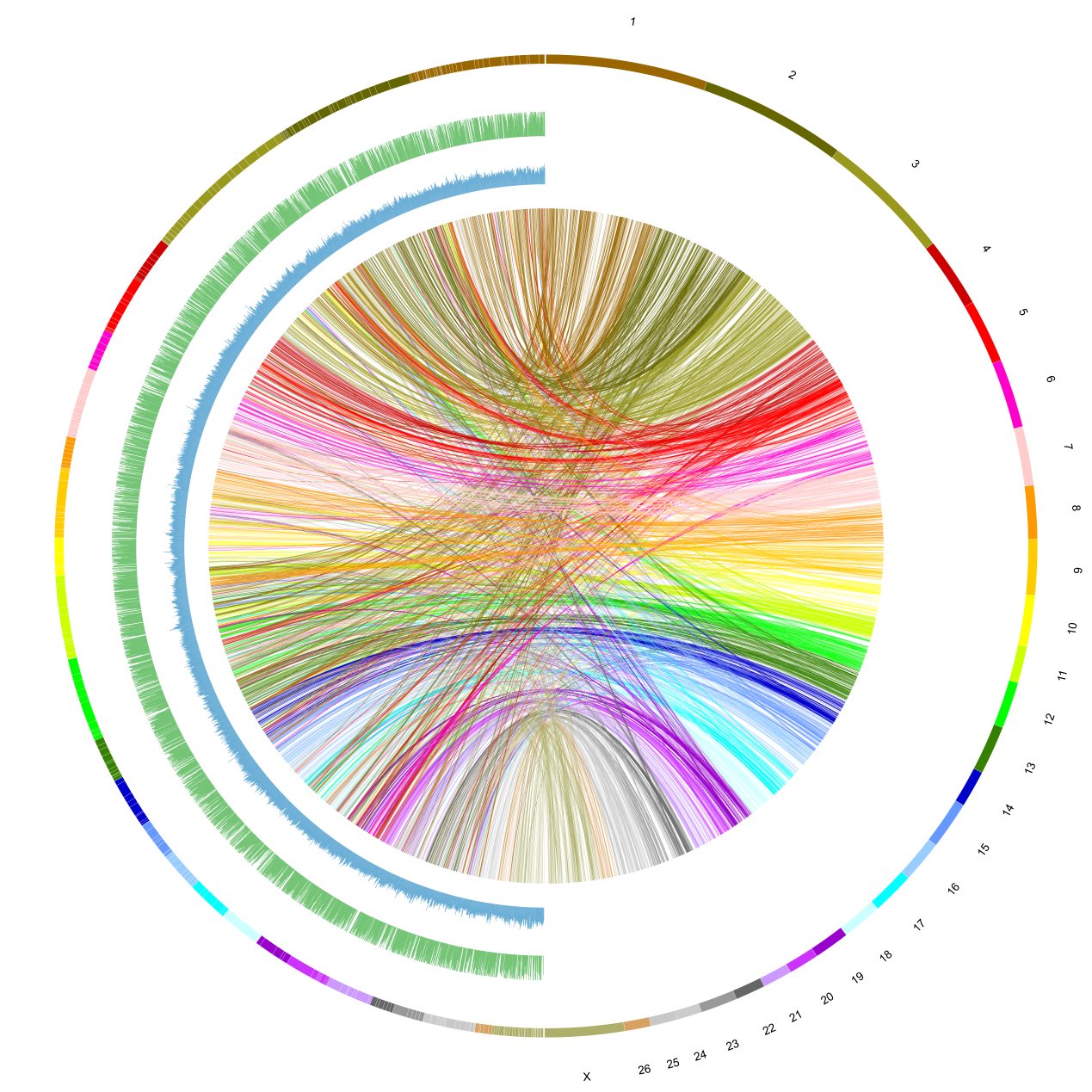
**

**Supplementary Figure 7.** **Synteny** **relationship between mouflon and sheep. The left part from outer circle to the inner circle, represent the mouflon scaffold, gene density (in 100kb windows) a nd repeat density (in 100kb windows) at the corresponding position respectively. The right part represents chromosome of sheep. The line between them represents the synteny relationship.**

**
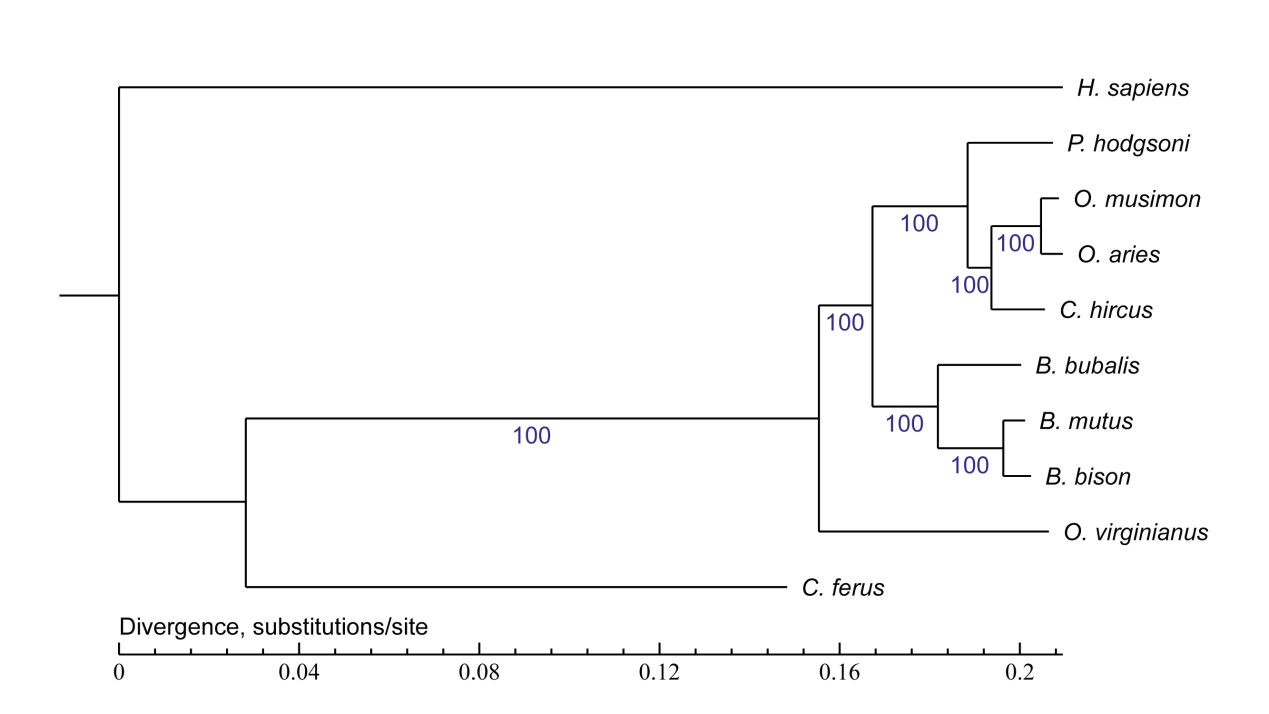
**

**Supplementary Figure 8a.** **Phylogenetic tree using all single-copy orthologs.**


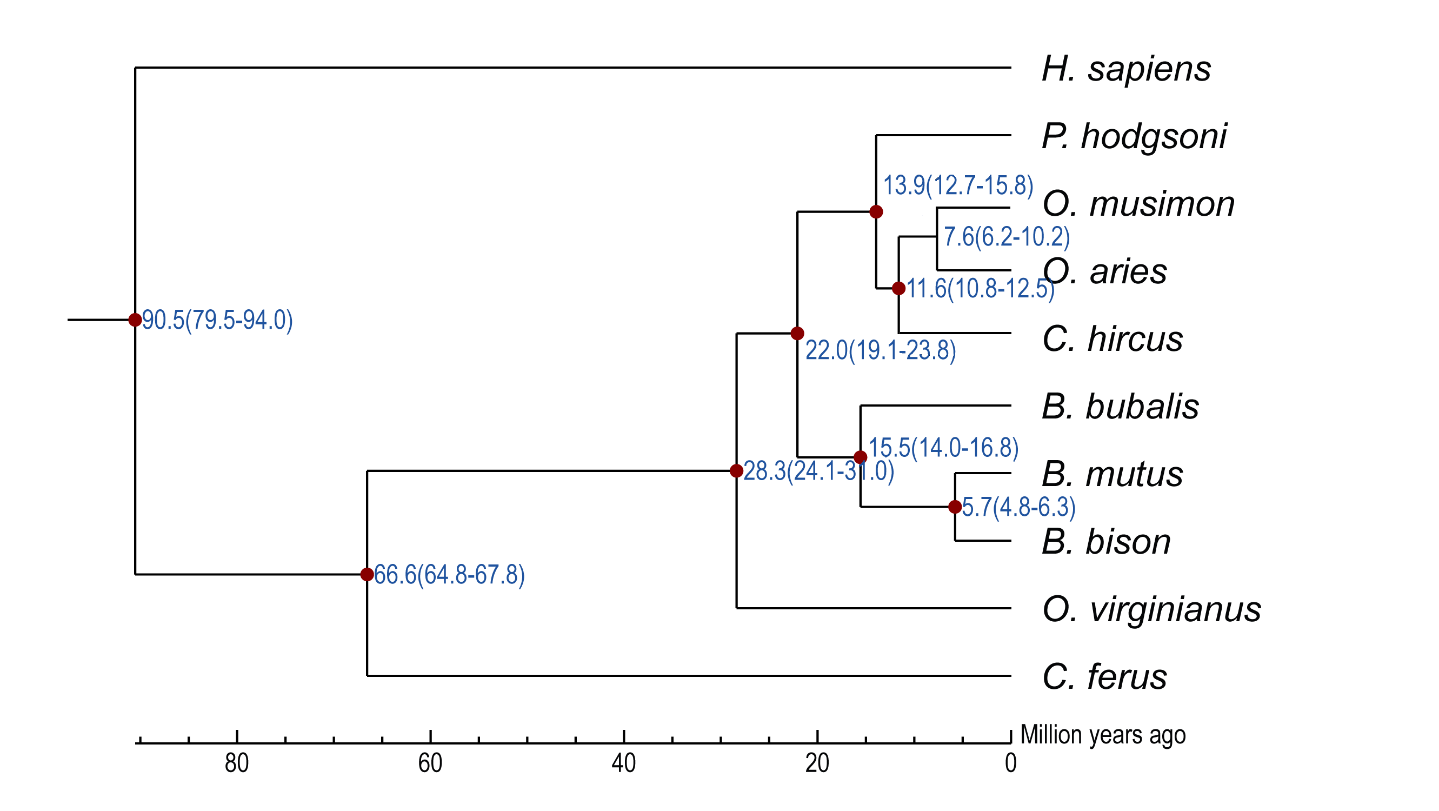


**Supplementary Figure 8b. Divergence time estimation.**


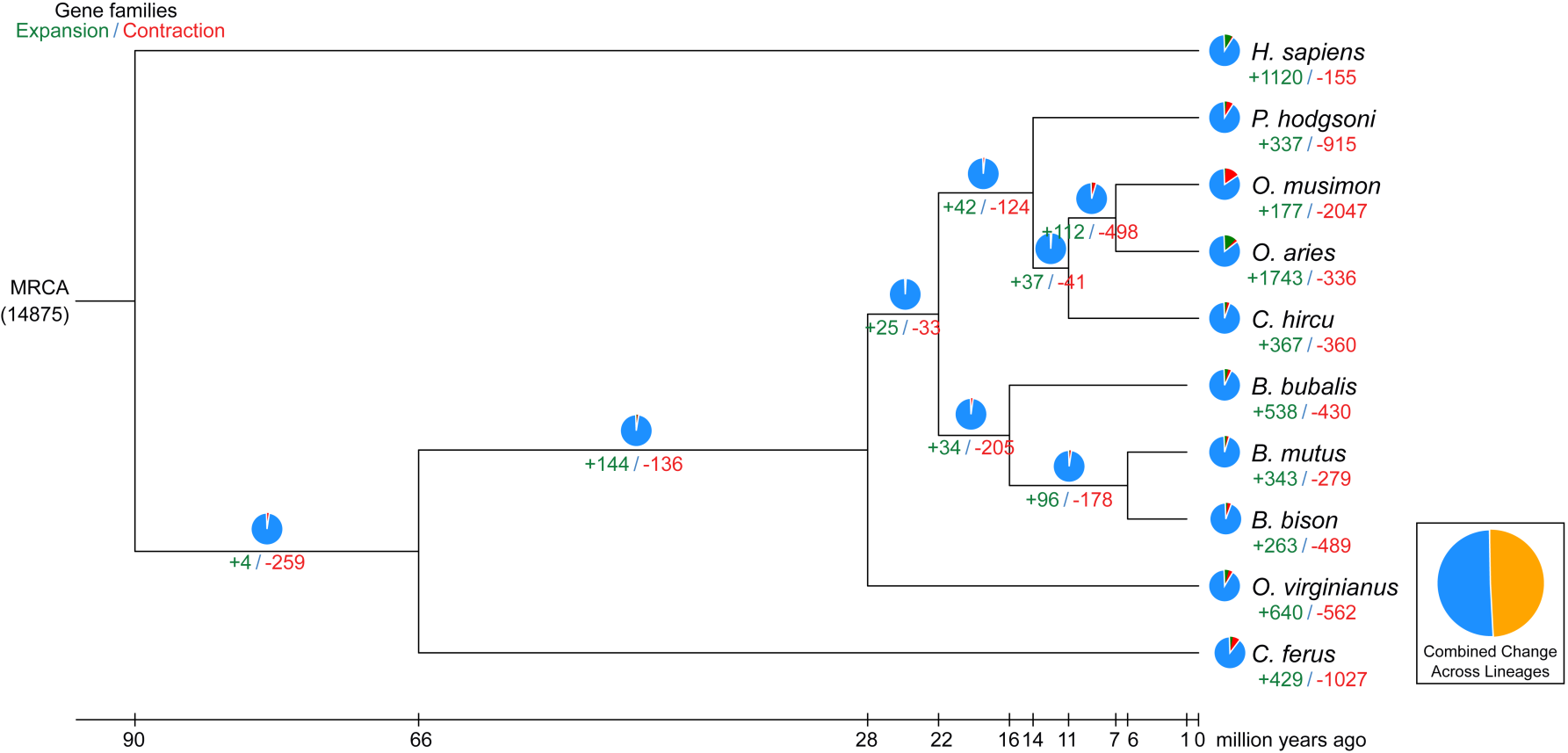


**Supplementary Figure 9. The distribution of gene families expansion and contraction.**

**
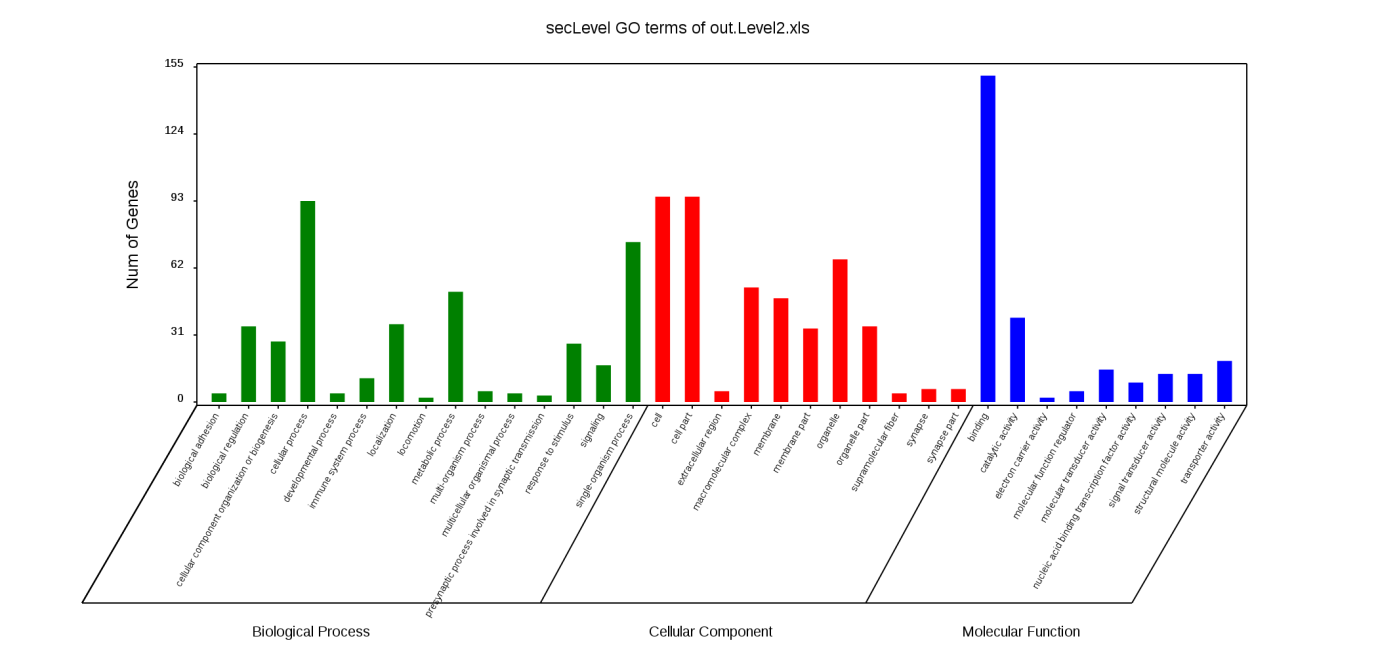
**

**Supplementary Figure 10. GO enrichment analysis of the expanded gene families in the mouflon.**


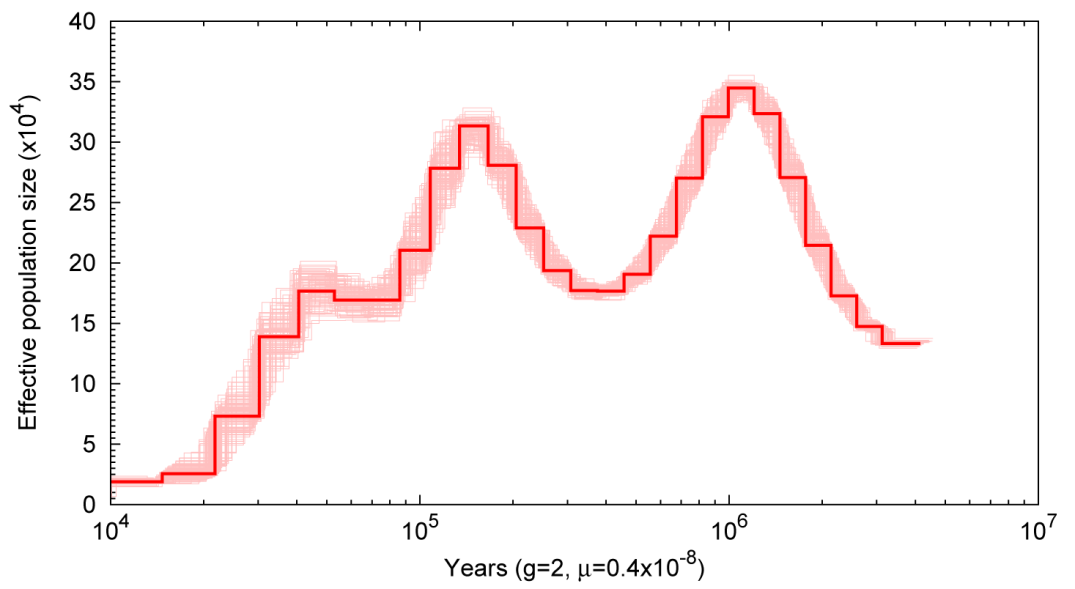


**Supplementary Figure 11. Demographic history of** **mouflon.**

**Supplementary Table 1. Statistics of raw data**

| Insert Size (bp) | Library ID | Raw Reads | Raw Length  (bp) | Raw Bases  (Gb) | Coverage | SRA number | GC content |
| --- | --- | --- | --- | --- | --- | --- | --- |
| 244 | GZA1-2-1 | 214 563 775 | 101 | 43.342 | 15.102 | SRR5520601 | 48.50% |
| 244 | GZA1-2-2 | 213 221 534 | 101 | 43.071 | 15.007 | SRR5520616 | 48.40% |
| 334 | GZA1-5-2 | 65 366 610 | 101 | 13.204 | 4.601 | SRR5520617 | 49.20% |
| 334 | GZA1-5-3 | 67 757 858 | 101 | 13.687 | 4.769 | SRR5520618 | 49.20% |
| 363 | GZA2-1-4 | 85 217 623 | 101 | 17.214 | 5.998 | SRR5520619 | 48.20% |
| 363 | GZA2-1-5 | 76 267 831 | 101 | 15.406 | 5.368 | SRR5520620 | 48.30% |
| 363 | GZA2-1-6 | 80 383 657 | 101 | 16.237 | 5.657 | SRR5520682 | 48.30% |
| 472 | GZA2-4-7 | 75 054 043 | 101 | 15.161 | 5.283 | SRR5521313 | 48.50% |
| 472 | GZA2-4-8 | 74 382 802 | 101 | 15.025 | 5.235 | SRR5521433 | 48.50% |
| 400 | DMY1 | 340 090 092 | 150 | 102.027 | 35.549 | SRR5520555 | 43.30% |
| 500 | DMY4 | 374 775 576 | 150 | 112.433 | 39.175 | SRR5520564 | 42.20% |
| 600 | DMY6 | 285 305 937 | 150 | 85.592 | 29.823 | SRR5520574 | 42.10% |
| 700 | DMY7 | 243 435 770 | 150 | 73.031 | 25.446 | SRR5520595 | 41.90% |
| 2000 | mM-2 | 209 645 451 | 101 | 42.348 | 14.755 | SRR5521447 | 43.40% |
| 5000 | M-4922 | 100 996 801 | 101 | 20.401 | 7.108 | SRR5521445 | 41.60% |
| 6000 | mM-6 | 193 707 515 | 101 | 39.129 | 13.634 | SRR5521448 | 43.20% |
| 10000 | M_10352 | 133 637 818 | 101 | 26.995 | 9.406 | SRR5521446 | 42.10% |
| 10000 | M_10 | 133 950 980 | 101 | 27.058 | 9.428 | SRR5521435 | 43.40% |
| 15000 | M_15-20k | 79 620 099 | 101 | 16.083 | 5.604 | SRR5521436 | 43.30% |
| 15000 | mM-10 | 213 676 540 | 101 | 43.163 | 15.039 | SRR5521450 | 43.40% |
| Total/Average | - | 3 261 058 312 | - | 780.607 | 271.989 | - | 45.35% |

**Supplementary Table 2. Statistics of clean data after filtering**

| Insert Size (bp) | Library ID | Clean Reads | Clean Length  (bp) | Clean Bases  (Gb) | Coverage |
| --- | --- | --- | --- | --- | --- |
| 244 | GZA1-2-1 | 196715051 | 100 | 39.343 | 13.708 |
| 244 | GZA1-2-2 | 195234177 | 100 | 39.047 | 13.605 |
| 334 | GZA1-5-2 | 61920886 | 100 | 12.384 | 4.315 |
| 334 | GZA1-5-3 | 64174130 | 100 | 12.835 | 4.472 |
| 363 | GZA2-1-4 | 80002081 | 100 | 16.000 | 5.575 |
| 363 | GZA2-1-5 | 71803129 | 100 | 14.361 | 5.004 |
| 363 | GZA2-1-6 | 75579750 | 100 | 15.116 | 5.267 |
| 472 | GZA2-4-7 | 70996429 | 100 | 14.199 | 4.947 |
| 472 | GZA2-4-8 | 70353661 | 100 | 14.071 | 4.903 |
| 400 | DMY1 | 305914730 | 140 | 85.656 | 29.845 |
| 500 | DMY4 | 338175977 | 140 | 94.689 | 32.993 |
| 600 | DMY6 | 278195722 | 140 | 77.895 | 27.141 |
| 700 | DMY7 | 237822008 | 140 | 66.590 | 23.202 |
| 2000 | mM-2 | 176954319 | 101 | 35.745 | 12.455 |
| 5000 | M-4922 | 91422262 | 100 | 18.284 | 6.371 |
| 6000 | mM-6 | 135748439 | 101 | 27.421 | 9.554 |
| 10000 | M_10352 | 117151097 | 100 | 23.430 | 8.164 |
| 10000 | M_10 | 114502984 | 100 | 22.901 | 7.979 |
| 15000 | M_15-20k | 57247275 | 100 | 11.449 | 3.989 |
| 15000 | mM-10 | 95669598 | 101 | 19.325 | 6.733 |
| Total | - | 2835583705 | - | 660.742 | 230.224 |

**Supplementary Table 3. Statistics of 21-mer analysis**

| Kmer size | Kmer num | Peak depth | Genome size | Used base | Used reads |
| --- | --- | --- | --- | --- | --- |
| 21 | 278426024880 | 97 | 2870371390 | 324830362360 | 2320216874 |

**Supplementary Table 4. Statistics of the completeness of the hybrid de novo assembly genome of by BUSCO**

|  | Gene numbers | Percentage |
| --- | --- | --- |
| Complete BUSCOs | 2477 | 95.8% |
| Complete and single-copy BUSCOs | 2418 | 93.5% |
| Complete and duplicated BUSCOs | 59 | 2.3% |
| Fragmented BUSCOs | 49 | 1.9% |
| Missing BUSCOs | 60 | 2.3% |
| Total BUSCO groups searched | 2586 | - |

**Supplementary Table 5. Statistic of reads aligned to mouflon assembly genome**

| Library ID | Insert Size (bp) | PE Total Reads | PE Mapped Reads | Ratio(%) |
| --- | --- | --- | --- | --- |
| GZA1-2-1 | 244 | 390969854 | 389092833 | 99.52 |
| GZA1-2-2 | 244 | 388151508 | 386301515 | 99.52 |
| GZA1-5-3 | 334 | 127933424 | 127042089 | 99.30 |
| GZA1-5-2 | 334 | 123439688 | 122580490 | 99.30 |
| GZA2-1-4 | 363 | 159474106 | 158527117 | 99.41 |
| GZA2-1-5 | 363 | 143116454 | 142221410 | 99.37 |
| GZA2-1-6 | 363 | 150656596 | 149742199 | 99.39 |
| GZA2-4-7 | 472 | 141482992 | 140425450 | 99.25 |
| GZA2-4-8 | 472 | 140214200 | 139204752 | 99.28 |
| DMY1 | 400 | 598876222 | 594776567 | 99.32 |
| DMY4 | 500 | 665394200 | 661426143 | 99.40 |
| DMY6 | 600 | 508488092 | 503469795 | 99.01 |
| DMY7 | 700 | 430290280 | 425823242 | 98.96 |
| Total | - | 3968487616 | 3940633602 | 99.30 |

**Supplementary Table 6. TEs content in the assembled mouflon genome**

| Type | Repbase TEs | | TE proteins | | De novo | | Combined TEs | |
| --- | --- | --- | --- | --- | --- | --- | --- | --- |
|  | Length (bp) | % in genome | Length (bp) | % in genome | Length (bp) | % in genome | Length (bp) | % in genome |
| DNA | 55990336 | 2.063 | 6476578 | 0.239 | 29650698 | 1.093 | 59113888 | 2.178 |
| LINE | 731246112 | 26.944 | 536404649 | 19.765 | 760033035 | 28.005 | 897080766 | 33.055 |
| SINE | 244677237 | 9.016 | - | - | 70722271 | 2.606 | 251085809 | 9.252 |
| LTR | 93247947 | 3.436 | 14276845 | 0.526 | 83883958 | 3.091 | 126101907 | 4.646 |
| Other | 1304 | 0.000 | - | - | - | - | 1304 | 0.000 |
| Total | 1114254334 | 41.057 | 556909629 | 20.520 | 988979150 | 36.441 | 1202255796 | 44.299 |

**Supplementary Table 7.** **Prediction of repetitive elements in the assembled mouflon genome.**

| Type | Repeat Size （bp） | % of genome |
| --- | --- | --- |
| Trf | 37,017,512 | 1.364 |
| Repeatmasker | 1,114,254,334 | 41.057 |
| Proteinmask | 556,909,629 | 20.520 |
| De novo | 1,020,053,168 | 37.586 |
| Total | 1,245,566,092 | 45.895 |

**Supplementary Table 8. Number of all kinds of non-coding RNA**

| Type | | Copy(w) | Average length(bp) | Total length(bp) | % of genome |
| --- | --- | --- | --- | --- | --- |
| miRNA | | 482 | 89.622 | 43198 | 0.001592 |
| tRNA | | 535 | 75.951 | 40634 | 0.001497 |
| rRNA | rRNA | 114 | 93.456 | 10654 | 0.000393 |
|  | 18S | 87 | 97.770 | 8506 | 0.000313 |
|  | 28S | 23 | 84.826 | 1951 | 0.000072 |
|  | 5.8S | 2 | 56 | 112 | 0.000004 |
|  | 5S | 2 | 42.5 | 85 | 0.000003 |
| snRNA | snRNA | 1428 | 114.145 | 162999 | 0.006006 |
|  | CD-box | 299 | 93.779 | 28040 | 0.001033 |
|  | HACA-boc | 263 | 135.479 | 35631 | 0.001313 |
|  | splicing | 831 | 114.400 | 95066 | 0.003503 |

**Supplementary Table 9. Statistics for ortholog gene family**

| Species | Genes number | Genes in families | Unclustered genes | Family number | Unique families | Average genes per family |
| --- | --- | --- | --- | --- | --- | --- |
| *Bison* | 20634 | 19467 | 1167 | 15443 | 16 | 1.26 |
| *Bos mutus*, | 20493 | 19767 | 726 | 15595 | 7 | 1.27 |
| *Bubalus* | 21510 | 19732 | 1778 | 15558 | 39 | 1.27 |
| *Camelus ferus* | 19416 | 18517 | 899 | 14828 | 26 | 1.25 |
| *Capra hircus* | 20583 | 19954 | 629 | 15586 | 25 | 1.28 |
| *Odocoileus virginianus* | 21047 | 19852 | 1195 | 15511 | 11 | 1.28 |
| *Ovis aries* | 22693 | 21187 | 1506 | 15490 | 64 | 1.37 |
| *Pantholops hodgsonii* | 25456 | 23872 | 1584 | 16098 | 1041 | 1.48 |
| *Ovis orientalis musimon* | 20814 | 16973 | 3841 | 13987 | 33 | 1.21 |
| *Homo sapiens* | 22622 | 21892 | 730 | 15049 | 173 | 1.45 |

**Supplementary Table 10. Positively selected genes in the mouflon**

| **Mouflon gene ID** | **Gene symbol** | **Gene name** | **KO number** |
| --- | --- | --- | --- |
| Omusim00653.t1 | *ZBTB20* | zinc finger and BTB domain containing 20 | K10501 zinc finger and BTB domain-containing protein 20 |
| Omusim00757.t1 | *Iqsec3, sag* | IQ motif and Sec7 domain 3 | K12495 IQ motif and SEC7 domain-containing protein |
| Omusim00958.t1 | *BFSP1* | beaded filament structural protein 1, filensin | K10378 beaded filament structural protein 1, filensin |
| Omusim00998.t1 | NA | NA | NA |
| Omusim01125.t1 | *KLHL2* | kelch-like 2, Mayven (Drosophila) | K10443 kelch-like protein 2/3 |
| Omusim01343.t1 | *Ap2b1* | adaptor-related protein complex 2, beta 1 subunit | K11825 AP-2 complex subunit beta-1 |
| Omusim01505.t1 | NA | glutamine-rich 1 | K01886 glutaminyl-tRNA synthetase [EC:6.1.1.18] |
| Omusim01509.t1 | NA | NA | NA |
| Omusim01658.t1 | NA | soluble guanylyl cyclase beta 2 subunit-like | K12319 guanylate cyclase soluble subunit beta [EC:4.6.1.2] |
| Omusim01993.t1 | NA | NA | NA |
| Omusim02270.t1 | NA | NA | NA |
| Omusim02284.t1 | NA | keratin 13-like | K07604 type I keratin, acidic |
| Omusim02349.t1 | NA | G protein-coupled receptor 158 | K08469 G protein-coupled receptor 158 |
| Omusim02397.t1 | *NGFR, MGC148470* | nerve growth factor receptor | K02583 nerve growth factor receptor (TNFR superfamily member 16) |
| Omusim02487.t1 | *NEFL, MGC139791* | neurofilament, light polypeptide | K04572 neurofilament light polypeptide |
| Omusim02488.t1 | *NEFM* | neurofilament, medium polypeptide | K04573 neurofilament medium polypeptide (neurofilament 3) |
| Omusim02606.t1 | NA | similar to Collagen alpha-1(VI) chain | K06238 collagen, type VI, alpha |
| Omusim02662.t1 | *LTBP1* | latent transforming growth factor beta binding protein 1 | K08023 latent transforming growth factor beta binding protein |
| Omusim02806.t1 | *NFAT5* | nuclear factor of activated T-cells 5, tonicity-responsive | responsive; K04446 nuclear factor of activated T-cells, cytoplasmic, calcineurin-dependent |
| Omusim02853.t1 | *NTRK2, MGC140769, Trk-B* | neurotrophic tyrosine kinase, receptor, type 2 (EC:2.7.10.1) | K04360 neurotrophic tyrosine kinase receptor type 2 [EC:2.7.10.1] |
| Omusim02940.t1 | *ZNF565, MGC152155* | zinc finger protein 565 | K09228 KRAB domain-containing zinc finger protein |
| Omusim03075.t1 | NA | NA | NA |
| Omusim03311.t1 | NA | NA | NA |
| Omusim03376.t1 | NA | NA | NA |
| Omusim03419.t1 | *MAP1LC3B* | microtubule-associated protein 1 light chain 3 beta | K10435 microtubule-associated protein 1 light chain |
| Omusim03517.t1 | *SLC7A3* | solute carrier family 7 (cationic amino acid transporter, y+ system), member 3 | K13865 solute carrier family 7 (cationic amino acid transporter), member 3 |
| Omusim03519.t1 | *SLC7A3* | solute carrier family 7 (cationic amino acid transporter, y+ system), member 3 | K13865 solute carrier family 7 (cationic amino acid transporter), member 3 |
| Omusim03530.t1 | *NLRP3* | NLR family, pyrin domain containing 3 | K12800 NACHT, LRR and PYD domains-containing protein 3 |
| Omusim03546.t1 | *ZNF850, ZNF208* | zinc finger protein 850 | K09228 KRAB domain-containing zinc finger protein |
| Omusim03697.t1 | *SRCAP* | Snf2-related CREBBP activator protein | K11661 helicase SRCAP [EC:3.6.4.-] |
| Omusim03785.t1 | *FBXL7* | F-box and leucine-rich repeat protein 7 | K10273 F-box and leucine-rich repeat protein 7 |
| Omusim04060.t1 | *DLG4, LLGL1* | discs, large homolog 4 (Drosophila) | K11828 discs large protein 4 |
| Omusim04074.t1 | *TNK1, Ack-like* | tyrosine kinase, non-receptor, 1 | K08885 non-receptor tyrosine-protein kinase TNK1 [EC:2.7.10.2] |
| Omusim04332.t1 | *WASF3* | WAS protein family, member 3 | K06083 WAS protein family, member 3 |
| Omusim04390.t1 | *KLHL4* | kelch-like 4 | K10442 kelch-like protein 1/4/5 |
| Omusim04758.t1 | NA | similar to Stromal antigen 1 | K05643 ATP-binding cassette, subfamily A (ABC1), member 3 |
| Omusim04808.t1 | NA | NA | NA |
| Omusim04900.t1 | *APOL3* | apolipoprotein L, 3 | K14480 apolipoprotein L |
| Omusim05280.t1 | *RFWD3* | ring finger and WD repeat domain 3 | K08849 mixed lineage kinase domain-like [EC:2.7.11.1] |
| Omusim05421.t1 | NA | MGC66101, zgc:85904; zgc:66101 (EC:2.7.11.1) | K08282 non-specific serine/threonine protein kinase [EC:2.7.11.1] |
| Omusim05491.t1 | *GRM1* | glutamate receptor, metabotropic 1 | K04604 metabotropic glutamate receptor 1/5 |
| Omusim05565.t1 | *VNN1* | vanin 1 (EC:3.5.1.92) | K08069 pantetheine hydrolase [EC:3.5.1.92] |
| Omusim06000.t1 | NA | similar to heterogeneous nuclear ribonucleoprotein H2 | K12898 heterogeneous nuclear ribonucleoprotein F/H |
| Omusim06150.t1 | *KIF13A* | kinesin family member 13A | K10392 kinesin family member 1/13/14 |
| Omusim06357.t1 | *C4BPA, MGC128750* | complement component 4 binding protein, alpha | K04002 complement component 4 binding protein, alpha |
| Omusim06370.t1 | NA | NA | NA |
| Omusim06535.t1 | NA | similar to collagen, type II, alpha 1 | K06236 collagen, type I/II/III/V/XI, alpha |
| Omusim06820.t1 | *SEC31B* | SEC31 homolog B (S. cerevisiae) | K14005 protein transport protein SEC31 |
| Omusim06864.t1 | *Fmo5* | flavin containing monooxygenase 5 (EC:1.14.13.8) | K00485 dimethylaniline monooxygenase (N-oxide forming) [EC:1.14.13.8] |
| Omusim07047.t1 | *AKR7A2, MGC148743* | aldo-keto reductase family 7, member A2 (aflatoxin aldehyde reductase) | K00540 [EC:1.-.-.-] |
| Omusim07117.t1 | *COL12A1* | collagen, type XII, alpha 1 | K08132 collagen, type XII, alpha |
| Omusim07346.t1 | NA | NA | NA |
| Omusim07858.t1 | NA | t-lymphoma invasion and metastasis-inducing protein 2-like | K05731 T-cell lymphoma invasion and metastasis |
| Omusim08008.t1 | *TNFRSF8* | tumor necrosis factor receptor superfamily, member 8 | K05145 tumor necrosis factor receptor superfamily member 8 |
| Omusim08336.t1 | NA | NA | NA |
| Omusim08770.t1 | NA | similar to multiple PDZ domain protein | K06095 multiple PDZ domain protein |
| Omusim08777.t1 | *ZFYVE20* | zinc finger, FYVE domain containing 20 | K12481 rabenosyn-5 |
| Omusim08883.t1 | *ICAM1* | intercellular adhesion molecule 1 | K06490 intercellular adhesion molecule 1 |
| Omusim08922.t1 | NA | mecom-a, MGC197023, evi-1, evi1, mecom, xEvi-1; MDS1 and EVI1 complex locus | K04462 ecotropic virus integration site 1 protein |
| Omusim09133.t1 | *FOXG1* | forkhead box G1 | K09385 forkhead box protein G |
| Omusim09162.t1 | NA | beta-galactoside alpha-2,6-sialyltransferase 1-like | K00778 beta-galactoside alpha-2,6-sialyltransferase (sialyltransferase 1) [EC:2.4.99.1] |
| Omusim09270.t1 | *SNCAIP* | synuclein, alpha interacting protein | K04558 synuclein, alpha interacting protein (synphilin) |
| Omusim09330.t1 | *SF3A2, MGC138049* | splicing factor 3a, subunit 2, 66kDa | K12826 splicing factor 3A subunit 2 |
| Omusim09454.t1 | NA | heterogeneous nuclear ribonucleoprotein A2/B1 | K13158 heterogeneous nuclear ribonucleoprotein A2/B1 |
| Omusim09459.t1 | *HNRNPM, HNRPM* | heterogeneous nuclear ribonucleoprotein M | K12887 heterogeneous nuclear ribonucleoprotein M |
| Omusim09469.t1 | *Fbn1, AI536462, B430209H23, Fib-1, Tsk* | fibrillin 1 | K06825 fibrillin 1 |
| Omusim09835.t1 | *CD99* | CD99 molecule | K06520 CD99 antigen |
| Omusim10051.t1 | NA | similar to procollagen alpha 2(V) | K06236 collagen, type I/II/III/V/XI, alpha |
| Omusim10140.t1 | NA | NA | NA |
| Omusim10420.t1 | *PRSS8, DKFZp469E0915* | protease, serine, 8 | K08664 protease, serine, 8 (prostasin) [EC:3.4.21.-] |
| Omusim10786.t1 | *ZBTB41* | zinc finger and BTB domain containing 41 | K10513 zinc finger and BTB domain-containing protein 41 |
| Omusim10848.t1 | *CENPF* | centromere protein F, 350/400kDa (mitosin) | K11499 centromere protein F |
| Omusim11021.t1 | NA | hypothetical protein | K14790 nucleolar protein 9 |
| Omusim11310.t1 | *AP3B2* | adaptor-related protein complex 3, beta 2 subunit | K12397 AP-3 complex subunit beta |
| Omusim11369.t1 | NA | cubilin, putative (EC:3.4.24.19 | K14616 cubilin |
| Omusim12057.t1 | NA | NA | NA |
| Omusim12089.t1 | *PPP1R15A, MGC137534* | protein phosphatase 1, regulatory (inhibitor) subunit 15A | K14019 protein phosphatase 1 regulatory subunit 15A |
| Omusim12285.t1 | NA | NA | NA |
| Omusim12399.t1 | *AKR1C1, 2-ALPHA-HSD, 20-ALPHA-HSD, C9, DD1, DD1/DD2, DDH, DDH1, H-37, HAKRC, HBAB, MBAB, MGC8954* | aldo-keto reductase family 1, member C1 (dihydrodiol dehydrogenase 1; 20-alpha (3-alpha)-hydroxysteroid dehydrogenase) (EC:1.3.1.20 1.1.1.149 1.1.1.112) | K00089 3alpha-hydroxysteroid dehydrogenase (A-specific) [EC:1.1.1.213]; K00212 trans-1,2-dihydrobenzene-1,2-diol dehydrogenase [EC:1.3.1.20] |
| Omusim12417.t1 | *ADARB2* | adenosine deaminase, RNA-specific, B2 | K13194 double stranded RNA-specific editase B [EC:3.5.-.-] |
| Omusim12572.t1 | *RPS6KA2* | ribosomal protein S6 kinase, 90kDa, polypeptide 2 (EC:2.7.11.1) | K04373 p90 ribosomal S6 kinase [EC:2.7.11.1] |
| Omusim12654.t1 | NA | NA | NA |
| Omusim12691.t1 | *NCOA6* | nuclear receptor coactivator 6 | K14971 nuclear receptor coactivator 6 |
| Omusim12955.t1 | *MTFMT* | mitochondrial methionyl-tRNA formyltransferase (EC:2.1.2.9) | K00604 methionyl-tRNA formyltransferase [EC:2.1.2.9] |
| Omusim12985.t1 | NA | hypothetical protein | K10260 F-box and WD-40 domain protein 7 |
| Omusim13019.t1 | NA | NA | NA |
| Omusim13036.t1 | NA | hypothetical LOC462006 | K04943 potassium intermediate/small conductance calcium-activated channel subfamily N member 2 |
| Omusim13117.t1 | *MLL2* | myeloid/lymphoid or mixed-lineage leukemia 2 | K09187 histone-lysine N-methyltransferase MLL2 [EC:2.1.1.43] |
| Omusim13127.t1 | NA | NA | NA |
| Omusim13201.t1 | NA | keratin 6A-like | K07605 type II keratin, basic |
| Omusim13204.t1 | *KRT79* | keratin 79 | K07605 type II keratin, basic |
| Omusim13362.t1 | NA | NA | NA |
| Omusim13637.t1 | *CLEC4F* | C-type lectin domain family 4, member F | K10060 C-type lectin domain family 4, member F |
| Omusim13646.t1 | *XDH* | xanthine dehydrogenase (EC:1.17.1.4 1.17.3.2) | K00106 xanthine dehydrogenase/oxidase [EC:1.17.1.4 1.17.3.2] |
| Omusim13770.t1 | *ZNF169* | zinc finger protein 169 | K09228 KRAB domain-containing zinc finger protein |
| Omusim13776.t1 | *JHDM1D* | jumonji C domain containing histone demethylase 1 homolog D (S. cerevisiae) | K11445 JmjC domain-containing histone demethylation protein 1D/E/F |
| Omusim14410.t1 | NA | GA16721 gene product from transcript GA16721-RA | K06270 protein phosphatase 1 regulatory subunit 12A |
| Omusim14790.t1 | *PRKAR2B, PRKAR2, RII-BETA* | protein kinase, cAMP-dependent, regulatory, type II, beta (EC:2.7.11.1) | K04739 cAMP-dependent protein kinase regulator |
| Omusim14835.t1 | NA | membrane-associated guanylate kinase, WW and PDZ domain-containing protein 2-like | K05629 atrophin-1 interacting protein 1 |
| Omusim15389.t1 | NA | NA | NA |
| Omusim15416.t1 | *PRIM1* | primase, DNA, polypeptide 1 (49kDa) | K02684 DNA primase small subunit [EC:2.7.7.-] |
| Omusim15482.t1 | *mll3* | myeloid/lymphoid or mixed-lineage leukemia 3 | K09188 histone-lysine N-methyltransferase MLL3 [EC:2.1.1.43] |
| Omusim15624.t1 | *UPF3A* | UPF3 regulator of nonsense transcripts homolog A (yeast) | K14328 regulator of nonsense transcripts 3 |
| Omusim15735.t1 | NA | similar to muscleblind-like 2 | K14943 muscleblind |
| Omusim15836.t1 | NA | NA | NA |
| Omusim15971.t1 | NA | NA | NA |
| Omusim15980.t1 | *SIPA1, MGC102688, MGC17037, SPA1* | signal-induced proliferation-associated 1 | K08013 signal-induced proliferation-associated gene 1 |
| Omusim15982.t1 | NA | NA | NA |
| Omusim16033.t1 | *ADAM2* | ADAM metallopeptidase domain 2 | K06833 disintegrin and metalloproteinase domain-containing protein 2 [EC:3.4.24.-] |
| Omusim16176.t1 | NA | NA | NA |
| Omusim16280.t1 | NA | leukocyte immunoglobulin-like receptor, subfamily B, member 5-like; | K06512 leukocyte immunoglobulin-like receptor |
| Omusim16317.t1 | NA | olfactory receptor 5-like | K04257 olfactory receptor |
| Omusim16738.t1 | NA | hypothetical protein LOC100452994 | K14165 dual specificity phosphatase [EC:3.1.3.16 3.1.3.48] |
| Omusim16780.t1 | *LYZ2, MGC129123* | lysozyme C-2 (EC:3.2.1.17) | K01185 lysozyme [EC:3.2.1.17] |
| Omusim17106.t1 | *Brd1, 1110059H06Rik, AI316859, KIAA4191, MGC164733, mKIAA4191* | bromodomain containing 1 | K11349 bromodomain-containing protein 1 |
| Omusim17414.t1 | *TG* | thyroglobulin | K10809 thyroglobulin |
| Omusim17686.t1 | *OPLAH, 5-OPase* | 5-oxoprolinase (ATP-hydrolysing) (EC:3.5.2.9) | K01469 5-oxoprolinase (ATP-hydrolysing) [EC:3.5.2.9] |
| Omusim17696.t1 | *ERBB2IP* | erbb2 interacting protein | K12796 erbb2-interacting protein |
| Omusim17700.t1 | NA | NA | NA |
| Omusim17899.t1 | NA | NA | NA |
| Omusim18342.t1 | *DGKK* | diacylglycerol kinase, kappa | K00901 diacylglycerol kinase [EC:2.7.1.107] |
| Omusim18425.t1 | *USP6, HRP1, TRE17, TRE2, Tre-2, USP6-short* | ubiquitin specific peptidase 6 (Tre-2 oncogene) (EC:3.4.19.12) | K11837 ubiquitin carboxyl-terminal hydrolase 6/32 [EC:3.1.2.15] |
| Omusim18527.t1 | *CACNA1F* | calcium channel, voltage-dependent, L type, alpha 1F subunit | K04853 voltage-dependent calcium channel L type alpha-1F |
| Omusim18716.t1 | *GATA_3* | GATA binding protein 3 | K09182 GATA-binding protein 1/2/3 |
| Omusim18905.t1 | NA | ankyrin | K06867 |
| Omusim18943.t1 | NA | sodium- and chloride-dependent glycine transporter 1-like | K05042 solute carrier family 6 (neurotransmitter transporter, glycine) member 9 |
| Omusim19072.t1 | NA | NA | NA |
| Omusim19160.t1 | *METTL16, METT10D* | methyltransferase like 16 | K11393 methyltransferase [EC:2.1.1.-] |
| Omusim19338.t1 | *TRPM1* | transient receptor potential cation channel, subfamily M, member 1 | K04976 transient receptor potential cation channel subfamily M member 1 |
| Omusim19524.t1 | *FADS2* | fatty acid desaturase 2 | K10226 fatty acid desaturase 2 (delta-6 desaturase) [EC:1.14.19.-] |
| Omusim19768.t1 | NA | NA | NA |
| Omusim19911.t1 | *ABCA2* | ATP-binding cassette, sub-family A (ABC1), member 2 | K05642 ATP-binding cassette, subfamily A (ABC1), member 2 |
| Omusim19982.t1 | *Kcnt1, Slack, rSlo2* | potassium channel, subfamily T, member 1 | K04946 potassium channel subfamily T member 1 |
| Omusim20332.t1 | NA | similar to cadherin related 23 | K06813 cadherin 23 |
| Omusim20501.t1 | *EIF3A, EIF3S10, EIF6* | eukaryotic translation initiation factor 3, subunit A | K03254 translation initiation factor 3 subunit A |
| Omusim20620.t1 | NA | e3 ubiquitin-protein ligase TRIM11-like | K10650 tripartite motif-containing protein 11 [EC:6.3.2.19] |
| Omusim20724.t1 | NA | similar to neurogenic locus notch (notch) | K02599 Notch |
| Omusim20761.t1 | *ZNF79* | zinc finger protein 79 | K09228 KRAB domain-containing zinc finger protein |
| Omusim20799.t1 | NA | similar to template acyivating factor-I alpha | K11290 template-activating factor I |
| Omusim20803.t1 | NA | hypothetical protein | K11996 adenylyltransferase and sulfurtransferase |
| Omusim20868.t1 | *sat1a, MGC114142, zgc:114142* | spermidine/spermine N1-acetyltransferase 1a (EC:2.3.1.57) | K00657 diamine N-acetyltransferase [EC:2.3.1.57] |
| Omusim20917.t1 | *CALM, CALM2* | calmodulin-like (EC:2.7.11.19) | K02183 calmodulin |
| Omusim21094.t1 | *MGAM* | maltase-glucoamylase (alpha-glucosidase) | K12047 maltase-glucoamylase [EC:3.2.1.20 3.2.1.3] |
| Omusim21121.t1 | *TRB@, BV27, BV6-5, BV9* | T cell receptor beta locus | K10785 T-cell receptor beta chain V region |
| Omusim21235.t1 | *ACCN3* | amiloride-sensitive cation channel 3 | K04830 amiloride-sensitive cation channel 3 |
| Omusim21274.t1 | *PAXIP1* | PAX interacting (with transcription-activation domain) protein 1; | K14972 PAX-interacting protein 1 |
| Omusim21325.t1 | NA | similar to type IV collagen alpha 1 chain | K06237 collagen, type IV, alpha |
| Omusim21410.t1 | NA | NA | NA |
| Omusim21471.t1 | *HDAC7* | histone deacetylase 7 (EC:3.5.1.98) | K11408 histone deacetylase 7 [EC:3.5.1.98] |
| Omusim21517.t1 | *DCTN3, MGC142778* | dynactin 3 (p22) | K10425 dynactin 3 |
| Omusim21717.t1 | NA | NA | NA |
| Omusim21872.t1 | NA | hypothetical protein LOC100086245 | K03909 tissue factor pathway inhibitor |
| Omusim21981.t1 | NA | NA | NA |
| Omusim22264.t1 | *SIRPB1* | Tyrosine-protein phosphatase non-receptor type substrate 1-like | K06551 signal-regulatory protein |
| Omusim22543.t1 | NA | NA | NA |
| Omusim22651.t1 | NA | similar to eukaryotic translation elongation factor 1 alpha 1 | K03231 elongation factor 1-alpha |
| Omusim22817.t1 | NA | NA | NA |
| Omusim22999.t1 | NA | SH3 domain containing ring finger 1 | K12171 E3 ubiquitin-protein ligase SH3RF1 [EC:6.3.2.19] |
| Omusim23067.t1 | *UGT1A3* | UDP glucuronosyltransferase 1 family, polypeptide A3 | K00699 glucuronosyltransferase [EC:2.4.1.17] |
| Omusim23605.t1 | NA | NA | NA |
| Omusim23684.t1 | *ATP6* | ATP synthase F0 subunit 6 | K02126 F-type H+-transporting ATPase subunit a [EC:3.6.3.14] |
| Omusim23694.t1 | NA | dnaJ homolog subfamily B member 5-like | K09511 DnaJ homolog subfamily B member 5 |
| Omusim23701.t1 | NA | NA | NA |
| Omusim23903.t1 | *FLOT1, DKFZp469E0515* | flotillin 1 | K07192 flotillin |
| Omusim23907.t1 | NA | NA | NA |

**Supplementary Table 11. KEGG pathway analysis of the positively selected genes in the mouflon**

| Pathway  (Total 164) | Genes with pathway annotation (Total 78) | All genes with pathway annotation | | P value  (P<0.05) | Pathway ID |
| --- | --- | --- | --- | --- | --- |
| Protein digestion and absorption | 5 | 146 | 0.007819 | | ko04974 |
| AGE-RAGE signaling pathway in diabetic complications | 5 | 149 | 0.008501 | | ko04933 |
| Caffeine metabolism | 1 | 4 | 0.033464 | | ko00232 |
| cGMP - PKG signaling pathway | 5 | 220 | 0.038498 | | ko04022 |
| Oxytocin signaling pathway | 5 | 220 | 0.038498 | | ko04921 |
| ECM-receptor interaction | 4 | 155 | 0.042243 | | ko04512 |
| Renin secretion | 3 | 93 | 0.044098 | | ko04924 |

**Supplementary Table 12. Statistic of animal genome assembly comparison**

|  |  |  | Scaffold | | Contig | | |
| --- | --- | --- | --- | --- | --- | --- | --- |
| Species Name | **Assembly level** | **Assembled genome size (Gb)** | **N50 (Mb)** | **N50 number** | | **N50 (Kb)** | **N50 number** |
| *Ovis aries musimon* (mouflon)(Oori1) | Scaffold | 2.59 | 2.21 | 328 | | 39.7 | 17,191 |
| *Ovis ammon polii* (Marco Polo Sheep) | Scaffold | 2.71 | 5.49 | 131 | | 30.7 | 22,965 |
| *Ovis canadensis* (bighorn sheep)(ASM103953v1) | Chromosome | 2.59 | 100.19 | 27 | | 8.1 | 1,181,796 |
| *Ovis aries* (sheep)(Oar_v4.0) | Chromosome | 2.62 | 100.01 | 5,465 | | 150.5 | 48,481 |
| *Ovis aries musimon* (European mouﬂon) | Scaffold | 2.69 | 10.4 | 74 | | 110.1 | 6293 |
